# Banding cytogenetics of the Barbary partridge *Alectoris barbara* and the Chukar partridge *Alectoris chukar* (Phasianidae): a large conservation with Domestic fowl *Gallus domesticus* revealed by high resolution chromosomes

**DOI:** 10.3897/CompCytogen.v12i2.23743

**Published:** 2018-06-04

**Authors:** Siham Ouchia-Benissad, Kafia Ladjali-Mohammedi

**Affiliations:** 1 University of Sciences and Technology Houari Boumediene, Faculty of Biological Sciences, LBCM lab., Team: Genetics of Development. USTHB, PO box 32 El-Alia, Bab-Ezzouar, 16110 Algiers, Algeria

**Keywords:** Barbary partridge *Alectoris barbara*, chukar partridge *Alectoris chukar*, endemic species, banding cytogenetics, high resolution chromosomes, homologies, intrachromosomal rearrangements

## Abstract

The development of avian cytogenetics is significantly behind that of mammals. In fact, since the advent of cytogenetic techniques, fewer than 1500 karyotypes have been established. The Barbary partridge *Alectoris barbara* Bonnaterre, 1790 is a bird of economic interest but its genome has not been studied so far. This species is endemic to North Africa and globally declining. The Chukar partridge *Alectoris chukar* Gray, 1830 is an introduced species which shares the same habitat area as the Barbary partridge and so there could be introgressive hybridisation. A cytogenetic study has been initiated in order to contribute to the Barbary partridge and the Chukar partridge genome analyses. The GTG, RBG and RHG-banded karyotypes of these species have been described. Primary fibroblast cell lines obtained from embryos were harvested after simple and double thymidine synchronisation. The first eight autosomal pairs and Z sex chromosome have been described at high resolution and compared to those of the domestic fowl *Gallus domesticus* Linnaeus, 1758. The diploid number was established as 2n = 78 for both partridges, as well as for most species belonging to the Galliformes order, underlying the stability of chromosome number in avian karyotypes. Wide homologies were observed for macrochromosomes and gonosome except for chromosome 4, 7, 8 and Z which present differences in morphology and/or banding pattern. Neocentromere occurrence was suggested for both partridges chromosome 4 with an assumed paracentric inversion in the Chukar partridge chromosome 4. Terminal inversion in the long arm of the Barbary partridge chromosome Z was also found. These rearrangements confirm that the avian karyotypes structure is conserved interchromosomally, but not at the intrachromosomal scale.

## Introduction

The Barbary partridge *Alectoris barbara* Bonnaterre, 1790 (Phasianidae) is the only native partridge naturally present in Algeria. This North African endemic species is found not only from Morocco to Egypt, but also in Gibraltar, Sardinia and the Canary Islands (Cramp and Simmons 1980, Madge and McGowan 2002). The Barbary partridge is a nesting sedentary bird found in different ecosystems: Mediterranean (coastal dunes and Atlas Mountains), Steppic and Saharian. This common game bird is prized for its meat; hence its overhunting leads to declining population size in some areas. Although the Barbary partridge is listed as Least Concern on the IUCN Red List (2015) (International Union of Conservation of Nature), it is nevertheless protected by several conventions. Indeed, the Barbary partridge was placed on the regulated species list protected by the Convention on International Trade in Endangered Species of Wild Fauna and Flora (CITES): Bird instruction 79/409 (Annex I, II / 2, III / 1). This bird is also protected by the Convention on the Conservation of European Wildlife and Natural Habitats (Bern Convention). Furthermore, the Barbary partridge has also a national scope of protection in commercialisation of some bird species on the French territory. Decline of the native population is mainly due to predation, to poaching (despite the law prohibiting hunting since 1991) and habitat degradation due to mechanisation of farming and urban proliferation (Madge and McGowan 2002). In Morocco, observations have also shown a sharp decrease in Barbary partridge populations, which could become alarming in the long term (Maghnouj 1991). Other factors such as excessive use of pesticides, hikers and stray animals could also disrupt the smooth conduct of breeding. All these factors are also responsible for the decline of partridge populations in Europe (Tejedor et al. 2007, Randi 2008).

In addition, introduction of the exotic Chukar partridge *Alectoris chukar* Gray, 1830 could also lead to introgression in the wild genome of native partridge and could give rise to infertile descendants. In fact, hybridisation may occur when isolating mechanisms break down naturally or as a result of human activity as in the *Alectoris* partridges (Barbanera et al. 2011). Several studies have recorded cases of artificial genetic pollution of *Alectoris rufa* Linnaeus, 1758 and *Alectoris graeca* Meisner, 1804 by the *Alectoris chukar* genome (Randi et al. 2003, Barbanera et al. 2005, Barilani et al. 2007, Tejedor et al. 2007). The Barbary partridge is the most phylogenetically divergent taxon in the genus *Alectoris*, while *Alectoris chukar* is the most recent gamebird (Randi 1996, Randi and Lucchini 1998, Kimball et al. 1999). *Alectoris barbara* and *chukar* lineages split from an ancestral species about 6 million years ago, at the Miocene-Pliocene boundary (Voous 1976, Randi et al. 1992).

Preservation of this endemic species is a priority, which has led to a restocking programme with captive-reared Barbary partridge carried out by the Centre Cynégétique de Zéralda (36°42'06"N, 2°51'47"E). The goal of this project is to obtain strains able to reproduce in captivity, and formulate demographic monitoring after repopulation. Although the Barbary partridge is the main game-bird species in North Africa, scarce research has been reported and it concerns the reproduction and ecology of this species (Alaoui 1992, Akil and Boudedja 2001). However, recent genetic studies have established genetic tests aiming to identify hybrid individuals (Rodríguez-García and Galián 2014). Actually, the only classical cytogenetics data reported on *Alectoris* genus concern red-legged and Chukar partridges whose karyotypes have been described by use of conventional staining (Arruga et al. 1996, Babak et al. 2014, Ishishita et al. 2014).

The Barbary partridge *Alectoris barbara* like the domestic fowl *Gallus domesticus* Linnaeus, 1758 belongs to the ancestral order of Galliformes which includes the most avian species whose genomes have been analysed. In fact, the domestic fowl is the best described one because of its economic importance. It is considered as a reference in phylogenetics and comparative genomics and represents the only standardised bird karyotype (Ladjali-Mohammedi et al. 1999). As a typical avian genome, the karyotype of the domestic fowl has 39 pairs of chromosomes represented by 10 pairs of autosomal macrochromosomes (1–10 chromosomes), 28 pairs of microchromosomes (11–38) and one pair of sex chromosomes. The male is the homogametic sex ZZ (equivalent to human XX), whereas the female is the heterogametic sex ZW (equivalent to human XY) (Masabanda et al. 2004). Despite their small physical size, microchromosomes are characterised by high gene density, high GC content (McQueen et al. 1996) and an early replicating pattern compared to macrochromosomes (Schmid et al. 1989, Ponce de Leon et al. 1992, Burt 2002). Due to the presence of high number of near-undistinguishable microchromosomes, most bird karyotypes are partial and confined to a few macrochromosomes (Shibusawa et al. 2004). However, the use of chicken probes has allowed identification of several microchromosomes in some bird species (Fillon et al. 1998, Nie et al. 2015, Galkina et al. 2017, Kretschmer et al. 2018).

On the other hand, the chicken is the first avian genome to have been sequenced (Hillier et al. 2004), followed by the zebra finch *Taeniopygia guttata* (Warren et al. 2010) and Turkey *Meleagris gallopavo* (Dalloul et al. 2010). The chicken genome assembly Gallus_gallus-4.0 covered 1.03 Gb or 96% of the total genome size, including the sequence of the 10 macrochromosomes, 19 microchromosomes and sex chromosomes (Schmid et al. 2015). Recently, coverage was improved by a gain of 183 Mb and three microchromosomes (30, 31 and 33) in the Gallus_gallus-5.0 assembly. However, 138 Mb are not yet assigned to chromosomes (Warren et al. 2017). Rapid advances in genome assembly software and technologies as Next Generation Sequencing (NGS) allowed entire genome sequencing of more than 57 birds (Dalloul et al. 2010, Jarvis et al. 2014). Among these species, 42 were a part of the Genome 10K Project which aims to facilitate the sequencing and analysis of 10.000 vertebrate genomes (http://genome10k.soe.ucsc.edu) (Genome 10K Community of Scientists 2009). The Avian Phylogenomics Consortium announced in 2015 a great project called B10K (web.bioinfodata.org/B10K) to generate draft genome sequences for all the 10.476 avian species within the next five years (until 2020). All these sequencing data corroborate the exceptional stability of avian karyotypes (Shibusawa et al. 2002, Derjusheva et al. 2004, Shibusawa et al. 2004). Indeed, the occurrence of interchromosomal rearrangements in birds is a relatively rare event estimated to 1.25 per million years, which is considerably lower than in mammals (Zhao and Bourque 2009, Romanov et al. 2014, Zhang et al. 2014). It is assumed that interchromosomal reshuffling could be the result of an adaptive response and a cause or consequence of speciation (King 1995, Griffin et al. 2007, Romanov et al. 2014).

Although avian high resolution mapping is well advanced, reported cytogenetic studies are nevertheless partial and fewer than those of mammals despite great contribution of this discipline. In fact, classical and banding cytogenetics highlighted important features of avian karyotype as interchromosomal stability (Tegelstrôm and Ryttman 1981, Belterman and De Boer 1984, Christidis 1990, Shibusawa et al. 2004) and intrachromosomal reshuffling in some macrochromosomes (Stock and Bunch 1982, Griffin et al. 2007, Hooper and Price 2017). Banding cytogenetics has also elucidated the process of karyotypic evolution in some orders of bird (Dobigny et al. 2004, Shibusawa et al. 2004, Nishida et al. 2008) and even in mammals (Di-Nizo et al. 2017).

The aim of the present study is to describe the chromosomes of Barbary partridge *Alectoris barbara* and Chukar partridge *Alectoris chukar* at high resolution level with morphological and dynamic banding techniques. Comparison of partridges and chicken banding patterns has been conducted in order to estimate the degree of conservation and rearrangements of these species during speciation.

## Material and methods

### Biological material

Barbary and Chukar partridge embryos were obtained from the Centre Cynégétique de Zéralda during the laying period (March to June). Four Barbary partridge and four Chukar partridge embryos were sampled after 5–6 days incubation at 37 °C, and kept under the same temperature and hygrometry conditions in the Laboratoire de Génétique du Développement (Faculté des Sciences Biologiques, USTHB) until at least 12 days old.

### Cell cultures

Primary fibroblast cell cultures were harvested from 6 to 12 days old embryos. The embryos were cleared from their annexes and totally ground in a trypsine solution (0.05%, Sigma). Cell suspension were incubated at 41 °C with an estimate concentration of 3×10^6^ cells/ml in RPMI 1640 culture medium (20 mM HEPES, GIBCO) supplemented with 10% foetal calf serum (FCS, GIBCO), 1% L-Glutamine 200 mM (Sigma), 1% Penicillin, Streptomycin and Fungizone (Sigma). Trypsinisation of cells was realised to enhance division ability (adapted from Ladjali 1994, Ladjali et al. 1995).

### Synchronisation of cell cultures

In order to increase the yield of metaphases and prometaphases cells, cultures were synchronised with a simple and double thymidine block during the S phase (Dutrillaux and Couturier 1981, Hayes et al. 1993, Ladjali et al. 1995). Cells were blocked for the first time during 16–18h with thymidine (final concentration: 10mg/ml, Sigma), and rinsed 2×15 min with BSS+ (Hank’s Balanced Salt Solution containing 5.6% NaHCO_3_ and 2 mM CaCl_2_) at 41 °C. Cells were incubated again in culture medium RPMI, and the day after, the step above was repeated for a second time to produce a double thymidine block. On the third day, when cells restarted division in RPMI with 5% FCS, an analogue of thymidine 5-Bromo-2-deoxyUridine (final concentration: 10 μg/ml, BrdU, Sigma) was incorporated into cultures. An hour after, 5-Fluoro-2-uridine (final concentration: 0.5 μg/ml, FdU, Sigma) was added to enhance BrdU incorporation. These treatments are required to prepare chromosomes for dynamic R-banding staining (Dutrillaux and Couturier 1981, Schmid et al. 1989, Hayes et al. 1993, Ladjali et al. 1995).

### Cell harvest

The incorporation of BrdU into the S phase lasted 6–7 hours. Meanwhile cells were continuously observed by reversed microscope until the number of mitotic round cells peaked. Cells were trypsinysed (trypsine 0.05% + 0.02% EDTA, GIBCO) and harvested in a 15 ml tube with colchicine (final concentration: 0.05 μg/ml, Sigma). After centrifugation, hypotonic treatment was undertaken during 13 min at 37 °C with diluted newborn calf serum (1:5). Intracytoplasmic structures were prefixed with 1 ml of methanol/acetic acid (3:1) at 37 °C. Fixation was finally realised at 4 °C and after centrifugation, 1 ml was let in tubes until spreading. Slides were washed, rubbed and placed in cold water. A few drops from the cell suspension were spread at 10 cm of cold slide and left to dry until staining procedures occurred (adapted from Ladjali et al. 1995).

### Banding staining

**GTG-banding** (G-bands obtained with Trypsin and Giemsa) was realised following the Seabright modified method (1971). Approximately; 3 to 4 days after spreading, slides were incubated for 8–10 seconds in a trypsine solution (final concentration: 0.25%, Sigma) at room temperature. Slides were rinsed twice in PBS- (Phosphate Buffered Solution, pH=6.8) and stained in 6% Giemsa for 8–10 minutes.

**RBG-FPG banding** (R-bands followed by fluorochrome-photolysis) procedure was undertaken following Ladjali et al. (1995). Slides were incubated in Hoechst 33258 solution (1 mg/ml) for 20 min. Slides were then rinsed and placed for 90 min in 2 × SSC buffer (Saline Sodium Citrate) at a distance of 15 cm from UV dark light (Mazdafluor OE TFWN 20). Slides are rinsed again and placed in Earle’s buffer at 87 °C for 10 min. Slides were washed and incubated for 20 min in 6% Giemsa staining solution.

**RHG-banding** (R-bands obtained by Heat and Giemsa) was realised on *A. chukar* spreads. Slides were incubated in Earle’s buffer (ph=5,8) at 87 °C for 20 minutes, then rinsed and stained in 6% Giemsa solution (containing phosphate buffer) (Dutrillaux and Leujeune 1971, Comings 1978).

### Chromosome Classification

Slides were first observed with an optical microscope at objective magnification 10× to estimate the mitotic index (AxioZeiss Scope A1). Slides, showing a higher mitotic index, were analysed and prometaphases and metaphases, showing decondensed and dispersed chromosomes, were photographed (CoolCube1 Metasystems). The first eight macrochromosomes and Z sex chromosomes from Barbary partridge *Alectoris barbara* and Chukar partridge *Alectoris chukar* were classified in G- and R- banding as described in International System of Standardised Avian Karyotypes ISSAK (Ladjali-Mohammedi et al. 1999). Macrochromosomes pairs were classified according to decreasing size and centromere position (Shoffner 1974), whereas microchromosomes were not presented because of their small physical size making very difficult any classification or description. In order to avoid any ambiguity, nomenclature adopted in this article followed the ISSAK (Ladjali-Mohammedi et al. 1999) adapted from ISCN (1978).

### Chromosome measurement

Analyses measurements of fifteen first pairs of chromosomes were undertaken using KARYOTYPE 2.0 software (Altinordu et al. 2016). Measured parameters were: Long (q) and short (p) arms, total chromosome length (p+q) and arm ratio r: Long/short. In the Results section below, morphometry will be presented of the first eight chromosomes and the Z chromosome, which have been compared to the domestic fowl. Other microchromosomes were physically too small and did not give significant values. Partridge’s karyotypes have been established manually, considering that software used in the present work was not adapted to birds.

## Results

Primary fibroblasts cell lines were obtained a few hours after incubation and constituted a good source for obtaining chromosome preparations. The younger the embryos, the more mitotic divisions were obtained. The strict follow up of cell divisions after inhibition removal enabled the estimation of half cycle time to 7–8 hours for Barbary partridge *Alectoris barbara* and 6–7 hours for Chukar partridge *Alectoris chukar*. Important mitotic indices with high resolution chromosomes were obtained with simple synchronisation for *A. barbara* and double synchronisation for *A. chukar* during 18h. Furthermore, observation of cell cultures of both species showed that *A. barbara* cells were much more sensitive than *A. chukar* to the different drugs added during incubation. Trypsinisation and synchronisation steps caused important Barbary partridge cell death compared to Chukar partridge. In fact, we have incubated an average of 3×10^6^ cells/ml (Ladjali 1994). After a confluence, we estimate that cells have divided four times (12.10^6^ cells/ml). Following the trypsinisation, cells divided twice (24×10^6^ cells/ml). A continuous observation of cultures after *in vitro* treatments shows an average decrease of 30% of live cells of Barbary partridge, equivalent to 7.2×10^6^ cells/ml for all four embryos. Whereas, no diminution of mitotic power was observed in Chukar partridge regardless of trypsination, addition of BSS^+^, BrdU/FdU or colchicine.

**Figure 1.**
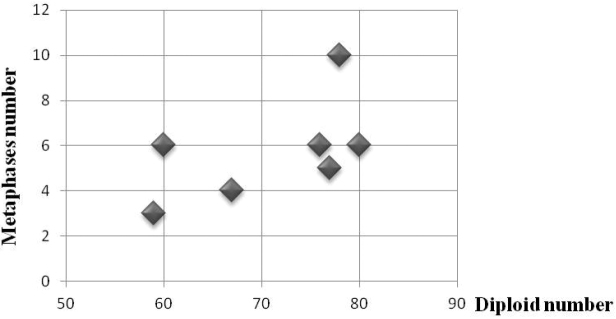
Estimation of diploid number of Barbary and Chukar partridges. Major metaphase plates (10) displayed diploid number 2n= 78 chromosomes.

Diploid numbers of Barbary partridge *Alectoris barbara* and Chukar partridge *Alectoris chukar* were estimated as 2n=78 from most metaphase plates (Fig. 1). Like most of birds, *A. barbara* and *A. chukar* karyotypes are composed of a few pairs of macrochromosomes and several microchromosomes with small physical size, which are very difficult to distinguish.

**Figure 2.**
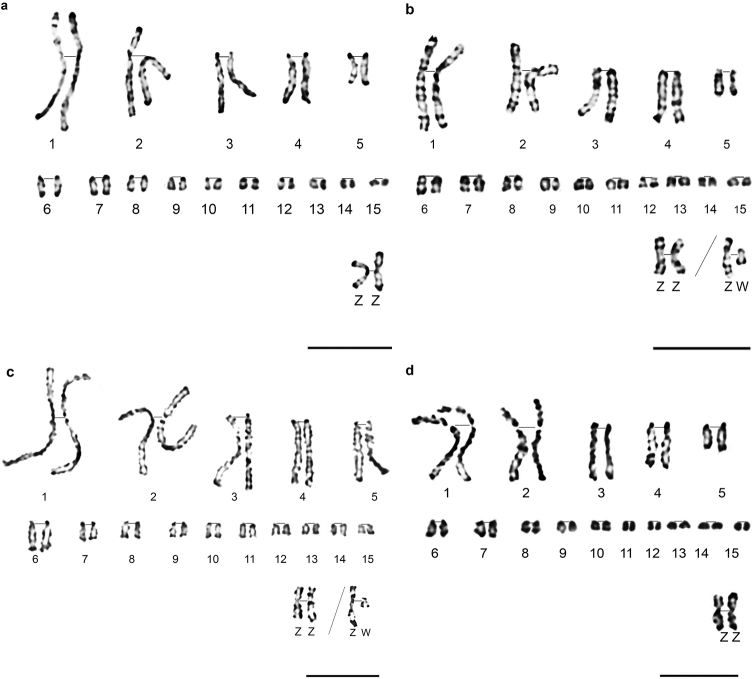
Partial karyotypes of *A. barbara* in GTG bands (**a**), *A. barbara* in RBG bands (**b**), *A. chukar* in GTG bands (**c**), and *A. chukar* in RHG bands (**d**). Gonosomes Z W are classified apart. Scale bars: 5 µm.

The authors proposed *Alectoris barbara* partial karyotype in GTG (Fig. 2a) and RBG banding (Fig. 2b), and *Alectoris chukar* partial karyotype in GTG (Fig. 2c) and RHG banding (Fig. 2d). Most metaphases show male genetic sex ZZ for both partridges, wherefore gonosome W was only described in RBG bands for *A. barbara* and GTG bands for *A. chukar*. The success of simple and double synchronisation resulted in high resolution chromosomes. Measurements show that chromosomes of *A. chukar* were more decondensed than those of *A. barbara* (Table 1). In fact, the size of the first eight macrochromosomes ranges from 14 *µ*m to 3*µ*m in *A. chukar* and from 9 *µ*m to 2 *µ*m for *A. barbara*. This is certainly due to the success of double synchronisation and extreme resistance of *A. chukar* cells to drugs added *in vitro*.

**Figure 3.**
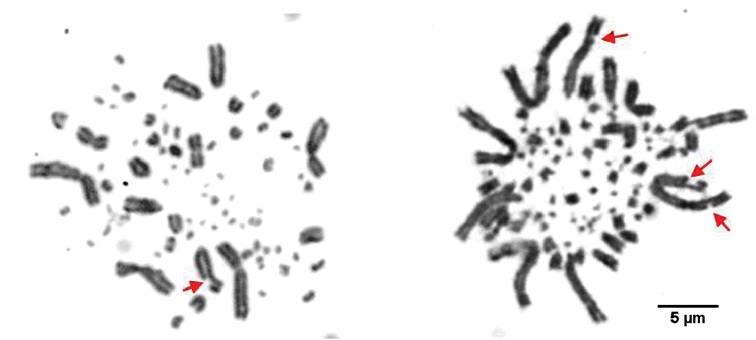
Partridges’ metaphases showing spatial distribution of chromosomes (*A. barbara* on the left and *A. chukar* on the right). Macrochromosomes are located towards metaphases periphery, microchromosomes are confined to the central area. *Arrows* indicates break points in chromosomes. Bar = 5 µm.

Observation of partridge’s spreads shows that in *A. barbara* an average of 45 metaphases /100 displayed break points. These breaks seem to appear in sub-terminal regions of macrochromosomes 1 and 3 (Fig. 3). None of *A. chukar* metaphases have shown this phenomenon. Furthermore, the same typical distribution of partridge’s chromosomes was observed. In fact, macrochromosomes are preferentially located towards the mitosis periphery, while microchromosomes are clustered within the mitosis interior (Fig. 3).

**Table 1.** *A. barbara* and *A. chukar* morphometry of the first eight macrochromosomes and gonosomes. Means are obtained at least from 10 prometaphases/metaphases (from 10 to 20). Chr: chromosome, q: long arm, p: short arm, t: total (p+q), r: ratio (q/p), lengths are given in micrometer (µm).

	*A. barbara*				*A. chukar*			
*Chr*	*p*	*q*	*t*	*r*	*p*	*q*	*t*	*r*
**1**	3.78	5.98	**9.76**	1.58	5.63	8.82	**14.45**	1.56
**2**	2.89	4.71	**7.6**	1.62	3.83	6.76	**10.59**	1.76
**3**	1.03	5.57	**6.6**	5.4	1.2	7.5	**8.7**	6.25
**4**	1.02	4.33	**5.35**	4.24	1.15	6.19	**7.34**	5.38
**5**	0.75	2.85	**3.6**	3.8	0.78	4.9	**5.68**	6.28
**6**	0.68	2.32	**3**	3.41	0.75	3.35	**4.1**	4.46
**7**	0.7	1.7	**2.4**	2.42	0.7	3	**3.7**	4.28
**8**	0.53	1.57	**2.1**	2.96	0.63	2.37	**3**	3.76
**Z**	2.5	3.1	**5.6**	1.24	3.1	3.5	**6.6**	1.12
**W**	0.75	1.03	**1.78**	1.37	0.93	1.37	**2.3**	1.47

**Figure 4.**
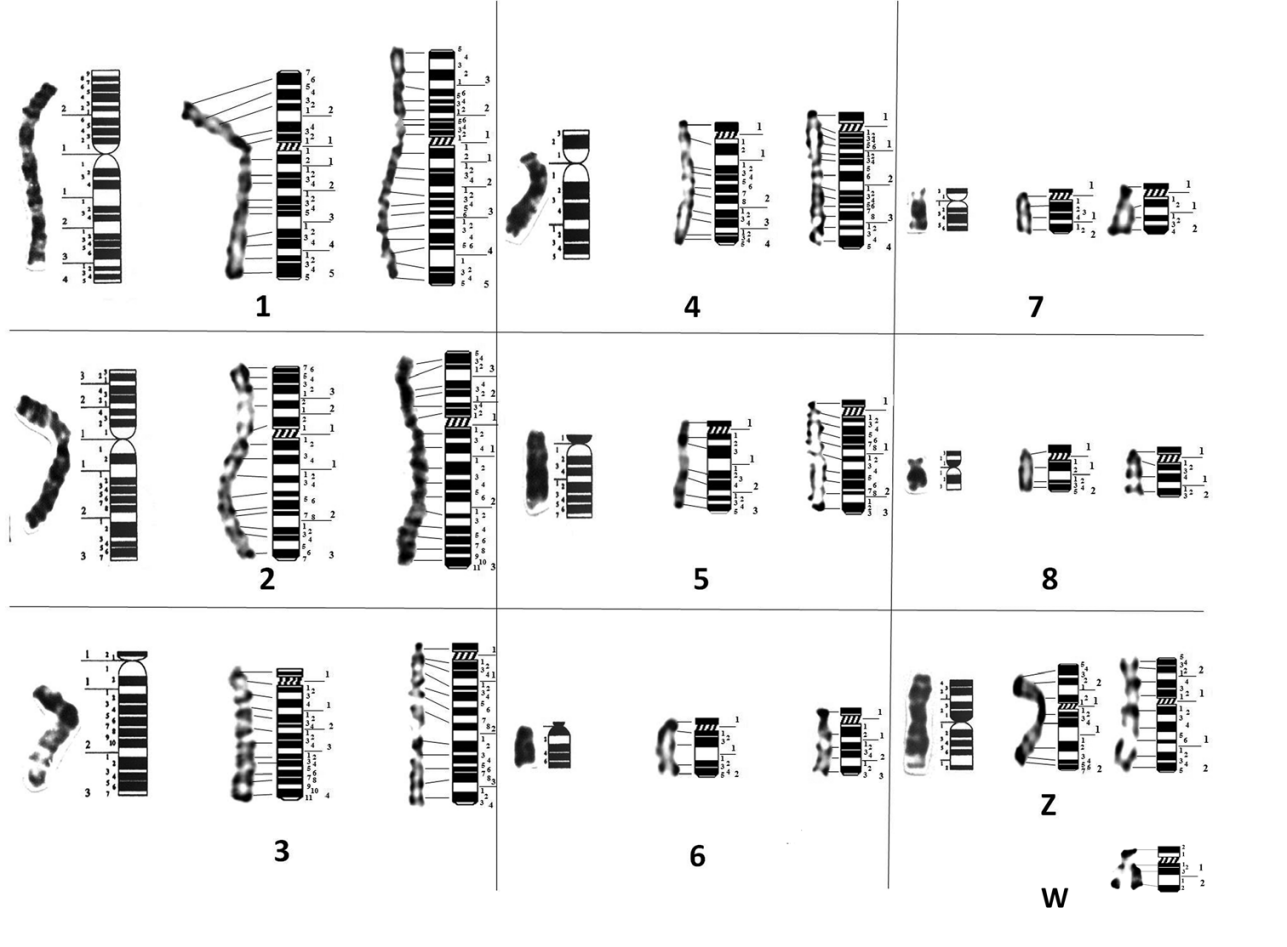
GTG partial ideograms of (from left to right) *G. domesticus* (Ladjali-Mohammedi et al. 1999), *A. barbara* and *A. chukar*. W chromosome is represented only in *A. chukar*. Horizontal traits indicate correspondence of positive bands between chromosomes and ideograms. Along ideograms: Large numbers indicate regions, smallest numbers indicate positive and negative bands.

**Figure 5.**
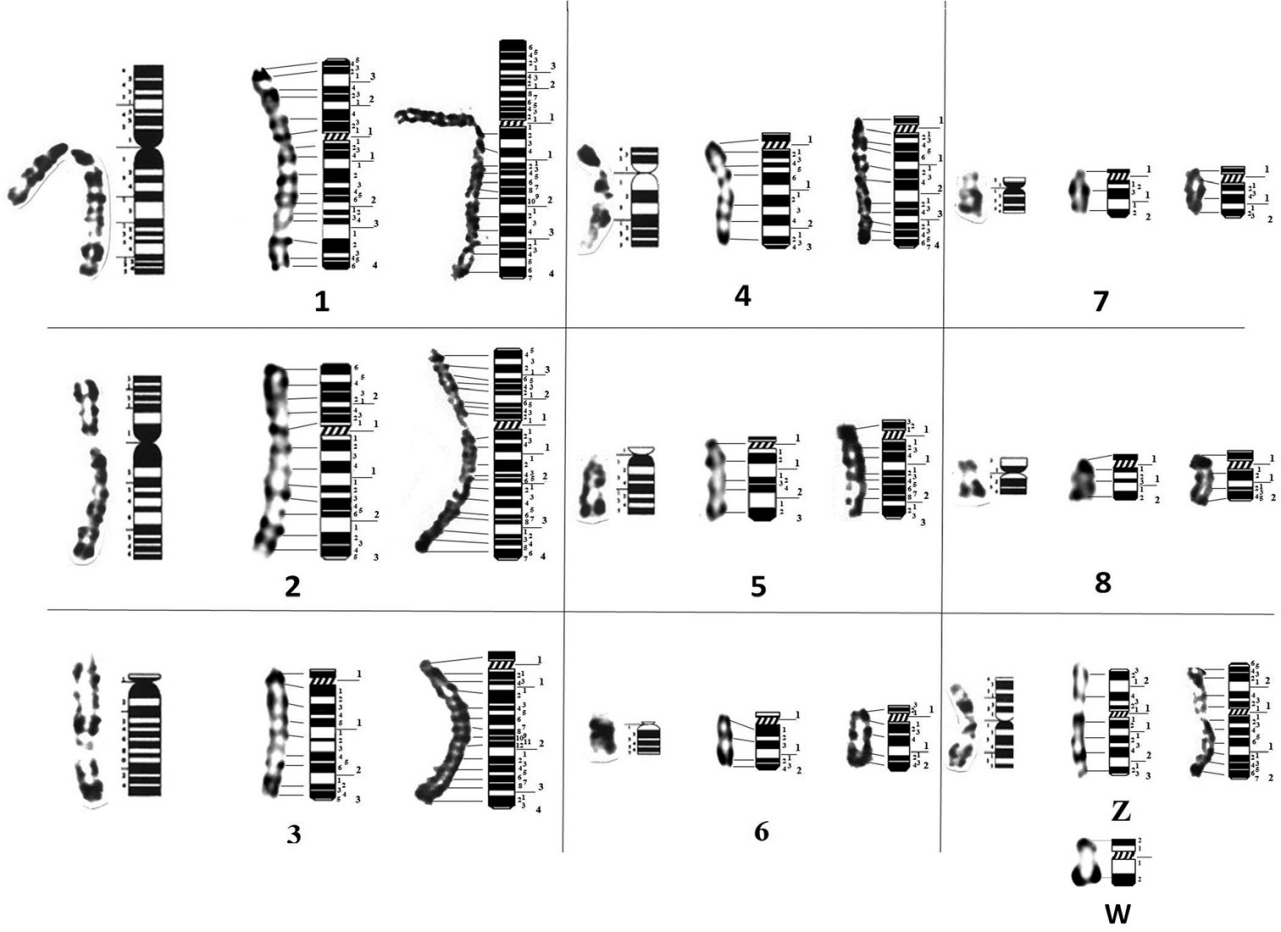
RBG and RHG partial ideograms of (from left to right) *G. domesticus* (Ladjali-Mohammedi et al. 1999), *A. barbara* and *A. chukar*. W chromosome is represented only in *A. barbara*. Horizontal traits indicate correspondence of positive bands between chromosomes and ideograms. Along ideograms: Large numbers indicate regions, smallest numbers indicate positive and negative bands.

Partial ideograms of *A. barbara* and *A. chukar* were proposed on the basis of means of 20 metaphases plates following the International System of Standardised Avian Karyotypes (Ladjali-Mohammedi et al. 1999) (Fig. 4 and Fig. 5, Table 2 and 3).

**Table 2.** Values summarized from partial ideograms of *A. barbara* and *A. chukar* described in GTG bands. Chr: chromosome, p: short arm, q: long arm, R: region, B: bands, LM: Landmark (all positions show negative landmarks except when (+) is added), empty boxes indicate that there is no particular landmark.

	*Alectoris barbara*						*Alectoris chukar*					
Chr	*P arm*			*Q arm*			*P arm*			*Q arm*		
	**R**	**B**	**LM**	**R**	**B**	**LM**	**R**	**B**	**LM**	**R**	**B**	**LM**
**1**	2	11	(21), (26)	5	21	(41)	3	17	(33)	5	23	(51)
**2**	3	11	(21)	3	19	(21), (31)	3	13	(31)	3	21	(31)
**3**	1	3	-	4	23	(13) (21)	1	2	-	4	23	(31), (41)
**4**	1	1	-	4	19	(21)	1	2	-	4	25	(26) +
**5**	1	1	-	3	12	(21)	1	2	-	3	19	(22) +
**6**	1	1	-	2	8	(21)	1	2	-	3	9	(22), (24)
**7**	1	1	-	2	6	-	1	1	-	2	6	(21)
**8**	1	1	-	2	7	-	1	1	-	2	7	-
**Z**	2	7	(21)	2	11	(21), (22)+	2	9	-	2	11	(15), (21)
**W**	-	-	-	-	-	-	1	2	-	2	5	(11)+(22)+

**Table 3.** Values summarized from partial ideograms of *A. barbara* and *A. chukar* described in RBG/RHG bands. Chr: chromosome, p: short arm, q: long arm, R: region, B: bands, LM: Landmark (all positions show negative landmarks except when (+) is added), empty boxes indicate that there is no particular landmark.

	*Alectoris barbara*						*Alectoris chukar*					
Chr	*P arm*			*Q arm*			*P arm*			*Q arm*		
	R	B	LM	R	B	LM	R	B	LM	R	B	LM
**1**	3	13	(31)	4	20	(41) (42)	3	18	(21) (31)	4	25	(13), (31), (45)
**2**	2	10	(25)	3	15	(21)	3	17	(21) (31)	4	25	(31)
**3**	1	2	-	3	16	(22)+(31)	1	2	-	4	27	(31)
**4**	1	2	-	3	14	(21) (31)	1	3	-	4	21	(14)+, (16)+ (22)+ , (24)+
**5**	1	2	-	3	8	(21) (31)	1	3	-	3	15	(12)+, (14)+
**6**	1	2	-	2	7	(21)	1	3	-	2	8	(21)
**7**	1	1	-	2	5	(21)	1	3	-	2	7	(14)+
**8**	1	1	-	2	5	(13)+	1	3	-	2	7	(21)
**Z**	2	7	(21)	3	9	(24)+, (31)	2	10	(21)	2	13	(21)
**W**	1	2	(12)+	1	2	(12)+	-	-	-	-	-	-

### Partial ideograms of *Alectoris barbara* and *Alectoris chukar* described in GTG bands (Fig. 4, Table 2)

***Chromosome 1***

***P arm***

Barbary partridge: two regions. 11 G bands with a visible negative band (21) which divides the p arm into two regions. A large terminal positive band is also visible (26).

Chukar partridge: three regions. 17 G bands with a predominant terminal negative band (33).

***Q arm***

Barbary partridge: Five regions. 21 bands, four negative bands divide the q arm into four regions with one predominant negative band (41). The centromeric region is positively banded.

Chukar partridge: Five regions. 23 G bands, with a wide terminal negative band (51).

***Chromosome 2***

***P arm***

Barbary partridge: three regions. 11 G bands with a large negative proximal band (21).

Chukar partridge: three regions. 13 G bands with large negative terminal band (31).

***Q arm***

Barbary partridge: three regions. 19 G bands with two wide negative bands (21 and 31).

Chukar partridge: three regions. 21 G bands with a large negative subtelomeric band (31).

**Chromosome 3**

***P arm***

Barbary partridge: one region with 3 G bands.

Chukar partridge: one region with 2 G bands.

***Q arm***

Barbary partridge: four regions. 23 G bands with two wide proximal negative bands (13 and 21).

Chukar partridge: four regions. 23 G bands with two large negative bands (31 and 41).

**Chromosome 4**

***P arm***

Barbary partridge: one region.

Chukar partridge: one region with 2 G bands.

***Q arm***

Barbary partridge: four regions. 19 G bands with a wide proximal negative band (21).

Chukar partridge: four regions. 25 G bands with a visible central positive band (26).

**Chromosome 5**

***Q arm***

Barbary partridge: three regions. 12 G bands with a wide central negative band (21).

Chukar partridge: three regions. 19 G bands with a visible central positive band (22).

**Chromosome 6**

***Q arm***

Barbary partridge: two regions. 8 G bands with a wide central negative band (21).

Chukar partridge: two regions. 9 G bands with two central positive bands (22 and 24).

**Chromosome 7**

***Q arm***

Barbary partridge: two regions. 6 G bands.

Chukar partridge: two regions. 6 G bands with a visible central negative band (21).

**Chromosome 8**

***Q arm***

Barbary partridge: two regions. 7 G bands with a wide central negative band (21).

Chukar partridge: two regions. 7 G bands with a large terminal negative band (21).

**Chromosome Z**

***P arm***

Barbary partridge: two regions. 7 G bands showing a large negative band (21).

Chukar partridge: two regions. 9 G bands with a visible negative band (21).

***Q arm***

Barbary partridge: two regions. 11 G bands with a large negative band (21) and a positive land mark (22).

Chukar partridge: two regions. 11 G bands with two large negative bands (15 and 21).

**Chromosome W**

***P arm***

Chukar partridge: one region. 2 G bands with terminal positive band.

***Q arm***

Chukar partridge: two regions. 5 G bands with one positive subcentromeric band (11) and a telomeric positive band (22)

### Partial ideograms of *Alectoris barbara* and *Alectoris chukar* described in RBG / RHG bands (Fig. 5, Table 3)

**Chromosome 1**

***P arm***

Barbary partridge: three regions. 13 RBG bands with a large terminal negative band (31).

Chukar partridge: Three regions. 18 RHG bands with two principal negative bands (21 and 31).

***Q arm***

Barbary partridge: Four regions. 20 bands with two wide terminal respectively negative and positive bands (41 and 42). The centromeric region is positively banded.

Chukar partridge: Four regions. 25 bands with three large negative bands which divided the q arm (13, 31 and 45).

**Chromosome 2**

***P arm***

Barbary partridge: two regions. 10 bands with a large negative telomeric band (25).

Chukar partridge: three regions. 17 bands with large negative proximal band (21).

***Q arm***

Barbary partridge: three regions. 15 bands with two wide negative bands (21 and 31).

Chukar partridge: four regions. 25 bands with a large negative telomeric band (31).

**Chromosome 3**

***P arm***

Barbary partridge: one region with 2 bands.

Chukar partridge: one region with 2 bands.

***Q arm***

Barbary partridge: three regions. 16 bands with a central positive band (22) and a telomeric negative band (31).

Chukar partridge: four regions. 27 bands with a large submedian negative band (31).

**Chromosome 4**

***P arm***

Barbary partridge: one region and 2 bands.

Chukar partridge: one region with 3 bands.

***Q arm***

Barbary partridge: three regions. 14 bands with two visible negative bands (21 and 31).

Chukar partridge: four regions. 21 bands with two proximal positive bands (14 and 16) and two central positive bands (22 and 24).

**Chromosome 5**

***P arm***

Barbary partridge: one region. 2 RBG bands.

Chukar partridge: one region. 3 RHG bands.

***Q arm***

Barbary partridge: three regions. 8 bands with two wide negative bands (21 and 31).

Chukar partridge: three regions. 15 bands with two large proximal positive bands (12 and 14).

**Chromosome 6**

***P arm***

Barbary partridge: one region showing 2 RBG bands.

Chukar partridge: one region presenting 3 RHG bands.

***Q arm***

Barbary partridge: two regions. 7 bands with a wide central negative band (21).

Chukar partridge: two regions. 8 bands and a large negative band (21).

**Chromosome 7**

***P arm***

Barbary partridge: one region.

Chukar partridge: one region with 3 RHG bands.

***Q arm***

Barbary partridge: two regions. 5 bands showing a large distal negative band (21).

Chukar partridge: two regions. 7 bands with a central positive band (14).

**Chromosome 8**

***P arm***

Barbary partridge: one region with one band.

Chukar partridge: one region with 3 RHG bands.

***Q arm***

Barbary partridge: two regions. 5 bands with a central positive band (13).

Chukar partridge: two regions. 7 bands and a central negative band (21).

**Chromosome Z**

***P arm***

Barbary partridge: two regions. 7 R bands and a wide terminal negative band (21).

Chukar partridge: two regions. 10 R bands with a large negative band (21).

***Q arm***

Barbary partridge: three regions. 9 R bands with a positive terminal land mark (24) and a large negative band (31).

Chukar partridge: two regions. 13 R bands with a visible terminal negative band (21).

**W chromosome**

***P arm***

Barbary partridge: one region. 2 RBG bands with terminal positive band.

***Q arm***

Barbary partridge: one region. 2 RBG bands with a large positive telomeric band. Centromeric region is negatively stained.

### *Alectoris barbara, Alectoris chukar* and *Gallus domesticus* chromosome comparison

**Figure 6.**
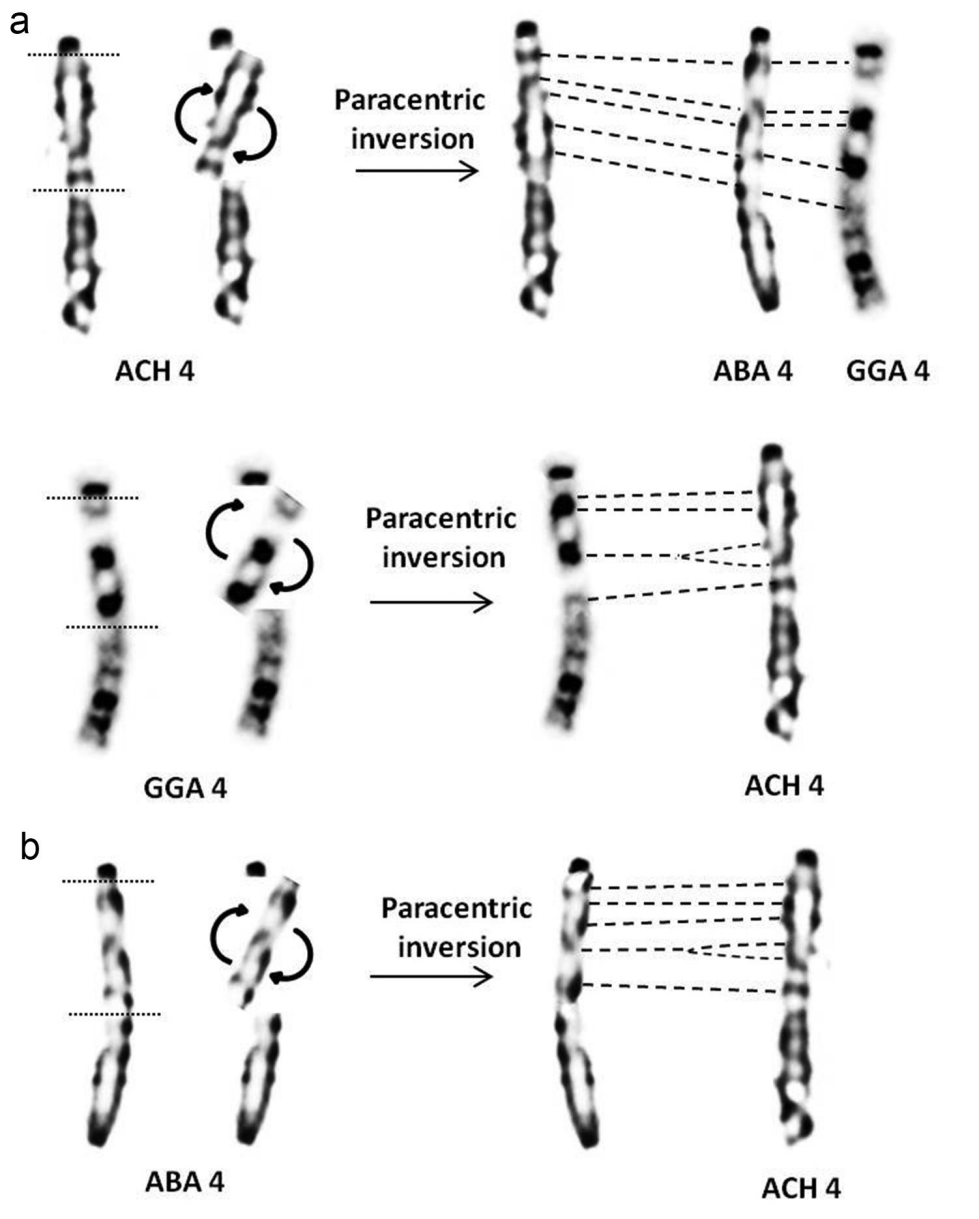
Schematic representation of a paracentric inversion in *A. chukar* (**a**), *G. domesticus* and *A. barbara* (**b**) chromosome 4. Corresponding bands are indicated by dashes. (ACH: *A. chukar*, GGA: *G. domesticus* and ABA: *A. barbara*).

**Figure 7.**
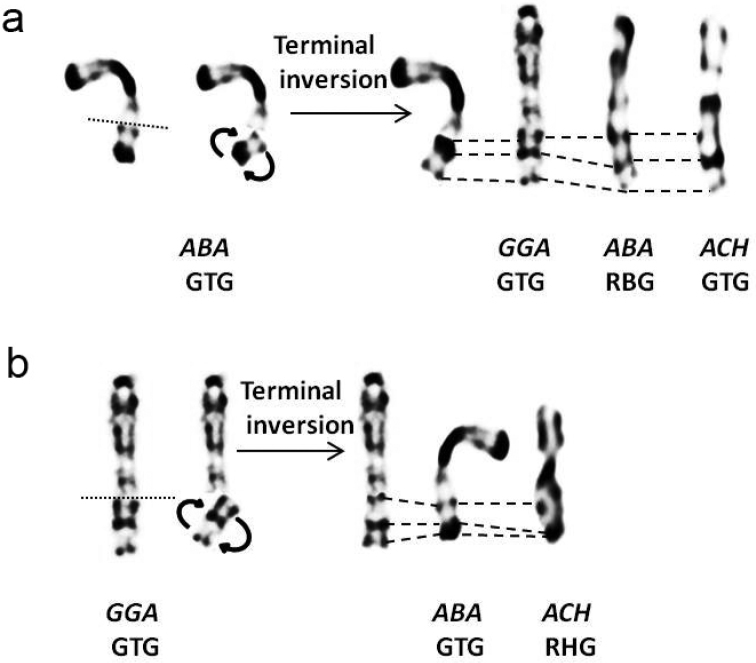
Schematic representation of a terminal paracentric inversion in chromosome Z of *A. barbara* in GTG (**a**) and chromosome Z of *G. domesticus* in GTG banding (**b**). Corresponding bands are indicated by dashes. (ACH: *A. chukar*, GGA: *G. domesticus* and ABA: *A. barbara*). Rearranged ABA Z in GTG corresponds to GGA Z and ACH Z in GTG. Rearranged GGA Z in GTG corresponds to ABA in GTG and ACH in RHG.

Comparison of morphological and dynamic G and R banding of *A. barbara* and *A. chukar* with domestic fowl (Ladjali-Mohammedi et al. 1999) shows a wide conservation of patterns in macrochromosomes. However, some rearrangements in partridges chromosomes 4 and Z were observed (Fig. 4 and Fig. 5). All centromeric regions of partridge chromosomes were positively stained in G and R banding. Chromosomes 1 and 2 are submetacentric in both Barbary and Chukar partridges, like in the domestic fowl. Despite the difference in chromosome 1 length, the arm ratio is quite similar (r = 1.58 / 1.56) (Table 1). On the other hand, the *A. chukar* long arm of chromosome 2 is longer than for *A. barbara* (r = 1.76 / 1.62). In both partridges, the centromere position is more submedian in chromosome 2 compared to chromosome 1. Chromosome 3 is acrocentric in partridges and domestic fowl. The banding pattern of the first three chromosomes is apparently widely conserved in all three species. Chromosome 4 is acrocentric in partridges and telocentric in chicken. The banding pattern is, however, conserved in *A. barbara* and *G. domesticus*, while in *A. chukar*, the subcentromeric region presents a different profile (Fig. 6). Chromosomes 5 and 6 are acrocentric in each species and present a similar pattern distribution, although, *A. chukar* presents higher number of bands due to decondensation. Chromosome 7 and 8 are acrocentric in both partridges and respectively, telocentric and submetacentric in the domestic fowl (Ladjali-Mohammedi et al. 1999). Surprisingly, the distribution of bands is conserved through these three Galliformes (comparison of chromosomes at the same decondensation stage). Sex chromosomes Z and W are submetacentric and morphologically conserved in all three species. However, the terminal region of the Z chromosome long arm presents a different pattern in *A. barbara* compared to that of *A. chukar* and the domestic fowl (Fig. 7). In the present work, we found in *A. barbara* a total of 145 G/123 R-bands and in *A. chukar* 173 G/187 R-bands only for the first eight chromosomes (Table 2 and 3).

## Discussion

Implementation of fibroblasts was observed in all cultures and confluence was quickly reached in all eight embryos, mainly in the youngest ones (6 days). This is due to the important mitotic power of cells at early embryonic stages (Ladjali et al. 1995). The high mortality in cell cultures of *A. barbara* (for the four embryos) is interestingly reflected in breeders’ observations regarding the Barbary partridge’s high vulnerability in breeding areas, unlike the Chukar partridge (personal communication of the Centre Cynégétique de Zéralda). Indeed, the Barbary partridge is a vulnerable endemic species, whereas Chukar partridge is usually introduced to reinforce the low local densities populations because of its easy practical prolificacy in captivity compared to other partridges (Rojas et al. 2011).

Distribution of partridges’ macrochromosomes and microchromosomes in metaphases is similar to that reported in several studies on chicken fibroblasts and neurons nucleis (Habermann et al. 2001, Federico et al. 2005) and mammalian fibroblasts nuclei (Cremer et al. 2000) (Fig. 3). In fact, it was reported that gene dense and early replicating chromatin, represented by microchromosomes (McQueen et al. 1996, Schmid et al. 1989, Ponce de Leon et al. 1992, Burt 2002, Skinner et al. 2009) were located in the nuclei central area, surrounded by gene-poor and later replicating chromatin (macrochromosomes) (Cremer et al. 2000). These results indicate that the radial position of chromosome territories is correlated with their size, their gene-density and replication timing (Habermann et al. 2001, Federico et al. 2005). Further, this specific distribution was assumed to be evolutionarily conserved in Galliformes (Maslova and Krasikova 2011) and also between mammals and birds despite their highly divergent karyotypes (Tanabe et al. 2002). The typical distribution of macro-and microchromosomes in metaphases could explain the particularly low rate of interchromosomal rearrangements in Galliformes (Shibusawa et al. 2002).

Fortuitously, 45% of *A. barbara* metaphase plates show breaks on some macrochromosomes which could be identified as fragile sites (Fig. 3). In birds, breakpoint regions of fragile sites are frequently associated with chromosomal rearrangements (Zlotina et al. 2010, Itoh et al. 2011, Skinner and Griffin 2012). Chromosomal fragile sites are loci prone to breakages within metaphase chromosomes (Fungtammasan et al. 2012). In mammals and birds, these breaks are assumed to occur in repetitive DNA clusters (Zlotina et al. 2010). Nevertheless, recent works in humans show that chromosomal rearrangements could appear in early replicating and actively transcribed gene clusters (Mortusewicz et al. 2013). It can be assumed that Barbary partridge chromosomes are particularly vulnerable to breakages, which could be favorable to intrachromosomal reshuffling. It would be very interesting to explore such genomic regions by molecular tools.

The diploid number of *Alectoris barbara* and *Alectoris chukar* was estimated as 2n = 78. This result is concordant with the exceptional stability of avian karyotype, i.e. about 65% of karyotyped birds displayed 76 to 82 chromosomes, including 7 to 8 pairs of macrochromosomes (Christidis 1990, Rodionov 1997). The diploid number of partridges emphasizes the conservation of karyotypes in the order of Galliformes (Stock and Bunch 1982, Shibusawa et al. 2002, Shibusawa et al. 2004). This is the case for the Chukar partridge described by Ishishita et al. (2014), as well as domestic fowl *Gallus domesticus* (Pollock and Fechheimer 1976, Ladjali-Mohammedi et al. 1999); Red-legged partridge *Alectoris rufa* (Arruga et al. 1996) and Japanese quail *Coturnix japonica* Temminck & Schlegel, 1849 (Stock and Bunch 1982). Interchromosomal conservation of partridges karyotype was also shown in previous studies. In fact, cross species painting using chicken macrochromosomes DNA probes (Zoo-FISH) has shown a perfect homology with, respectively, *A. chukar* and *Alectoris rufa* macrochromosomes (Kasai et al. 2003, Ishishita et al. 2014). Karyotypes of *A. barbara* and *A. chukar* show 8–10 pairs of macrochromosomes that have been measured and 30–28 pairs of microchromosomes whose morphology was difficult to determine despite obtaining high uncondensed chromosomes. Number of microchromosomes of partridges is quite similar to that of Galliformes (Stock and Bunch 1982, Shibusawa et al. 2002, Shibusawa et al. 2004). Microchromosomes were classified arbitrarily by decreasing size, their identification will be possible only by molecular cytogenetics (Zoo-FISH) using chicken microchromosomes specific markers (Fillon et al. 1998, Romanov et al. 2005).

Structural and dynamic R-bands obtained in the present work show similarities in pattern. However, dynamic RBG bands seem well delimited than morphological R-bands even if these latter present a higher number (Fig. 5). Pioneer studies have reported that RHG and RBG-bands are 75 to 85% congruent, and GTG and RHG-bands are 90% complementary, meaning that morphological G and R bands have a reverse pattern (Drouin et al. 1991). Dynamic and morphological R-bands are not totally stackable but correspond quite well and can be compared (Lemieux et al. 1990, Drouin et al. 1991).

Simple and double synchronisation of partridge cell cultures have offered the possibility to obtain important rate of prometaphasic chromosomes presenting high number of bands (Table 1 and 2). Comparatively, size of chicken macrochromosomes was ranged from 7 to 3 µm (Hammar 1966) and the first ten macrochromosomes of chicken haploid karyotype presented 209 G-bands and 182 R-bands (Ladjali et al. 1995). High resolution chromosomes allow detection of intrachromosomal changes that are not always visible at the metaphasic stage (Pollock and Fechheimer 1976, Ladjali et al. 1995, Ladjali-Mohammedi et al. 1999).

*A. barbara* and *A. chukar* chromosome 4 is acrocentric, while in *G. domesticus* it is telocentric. Furthermore, comparison of bands showed conservation of patterns in *A. barbara* and *G. domesticus* but not in *A. chukar*. This morphological difference could suggest repositioning of the centromere during the speciation event of partridges 6 million years ago (Randi et al. 1992). The difference in banding pattern in *A. chukar* could be explained by a paracentric inversion occurrence (4q11-4q31 in GTG) (Fig. 6). This result is supported by a previous study performed on red-legged partridge *A. rufa* chromosome 4, which is acrocentric (Arruga et al. 1996) and suggested that the morphological difference between *A. rufa* and *G. domesticus* was due to an inversion occurrence (Ramos et al. 1999). Later, Kasai et al. (2003) showed a perfect conservation of chicken BAC clones order on *A. rufa* chromosome 4 and introduced, for the first time in bird class, the term neocentromere (Kasai et al. 2003). Repositioning of the centromere or evolutionary new centromeres (ENC) is the movement of a centromere along the chromosome with the inactivation of the old one but without marker order alteration during evolution (Rocchi et al. 2012). Interestingly, this phenomenon is not so scarce and has been well described. In fact, several cases of *de novo* centromere formation have been reported in Japanese quail *Coturnix japonica* and Peking duck *Cairina moschata* Linnaeus, 1758 (Galkina et al. 2006, Skinner et al. 2009, Zlotina et al. 2012). Nevertheless, the hypothesis of double inversion occurrence should not be excluded as it was reported in the Japanese quail (Zlotina et al. 2012). High conservation of chromosome 4 in chicken and human over 300 million years has so far been reported (Chowdhary and Raudsepp 2000, Groenen et al. 2000). Conversely, the most common fusion reported in birds is between ancestral chromosome 4 and an ancestral microchromosome (Schmid et al. 2000, Shibusawa et al. 2002; 2004). In fact, in the chicken, whose karyotype is considered as the most similar to the ancestral bird karyotype, chromosome 4 is suggested to have arisen from a fusion of ancestral acrocentric chromosome 4 and ancestral microchromosome 10 (Belterman and De Boer 1984, Schmid et al. 2000, Griffin et al. 2007).

The morphological difference of chromosome 7 and 8 between partridges and the chicken, despite conservation of banding range, could be explained by repositioning of the centromere. However, double pericentric inversion cannot be excluded and only molecular investigations could elucidate such evolutionary events. Several studies show that chromosomes 7 and 8 are quite conserved in Galliformes and hybridize respectively to their homologous when using chicken chromosomal painting (Kasai et al. 2003). Exceptionally, in Guinea fowl *Numida meleagris* Linnaeus, 1758 belonging to Galliformes, Zoo-FISH with chicken DNA specific probes reveals a pericentric inversion in chromosome 7 which corresponds to chicken chromosome 8 (Shibusawa et al. 2002).

The Z chromosome in partridges shows a different terminal region. In fact, *A. barbara* Z gonosome presents an inversion of banding pattern in the terminus of long arm q compared to that of *A. chukar* and *G. domesticus*. Z gonosome of *A. barbara* in RBG corresponds to *G. domesticus* and *A. chukar* Z gonosome in GTG bands (Fig. 7). This result suggests occurrence of Z chromosome terminal inversion in the common ancestor of *A. barbara*, *G. domesticus* and *A. chukar* (Zq21 in GTG) (Fig. 7). The terminal region of Z chromosome in chicken is a characteristic heterochromatic band negatively stained in GTG (Ladjali-Mohammedi et al. 1999). Also, avian Z gonosome is particularly subjected to intrachromosomal rearrangements despite conservation of synteny in most species (Griffin et al. 2007, Nanda et al. 2008). In addition, total sequencing and assembly of chicken Z chromosome has confirmed low gene density (compared to autosomes) associated with high interspersed repeat content (Bellott et al. 2010), which is favorable to rearrangements (Völker et al. 2010).

In both partridges and chicken, the W chromosome is submetacentric and highly heterochromatic as reported in other studies on partridges (Ishishita et al. 2014, Arruga et al. 1996) (Fig. 2b, c). The W chromosome is ranked at the ninth position in *A. barbara* and *A. chukar* karyotypes. In different lineages of Neoaves, the W chromosome is supposed to have arisen by the accumulation of repetitive sequences and their conservation during evolution (Graves 2014, Schartl et al. 2016). A recent sequencing of chicken W chromosome has shown preservation of ancestral genes enriched for expressed dosage-sensitive regulators (Bellott et al. 2017). Therewith, it is well established that repetitive DNA polymorphism plays an important role in recombination, chromosomal instability and avian sex chromosome differentiation (Völker et al. 2010).

## Conclusion

Banding cytogenetics performed on high resolution chromosomes allowed the precise description of *Alectoris barbara* Bonnaterre, 1790 and *Alectoris chukar* karyotypes. Comparative chromosomal mapping highlighted a large conservation with domestic fowl *Gallus domesticus* Linnaeus, 1758. However, rearrangements in acrocentric macrochromosomes 4, 7 and 8 were observed. Except for the Z chromosome, the partridge chromosomes share more similarities with the putative Galliform ancestral karyotype (Belterman and De Boer, 1984) than with chicken. Such cytogenetic studies could be of an important contribution to detect eventual chromosomal rearrangements in hybrids, given that *A. barbara* and *A. chukar* share an overlapping area. Obviously, more detailed molecular cytogenetic studies are necessary to refine the results of the present work. Indeed, we have selected clones from Wageningen chicken BAC (Bacterial Artificial Chromosomes) library (Zoorob et al. 1996, Fillon et al. 1998, Crooijmans et al. 2000) and hybridized them on Barbary partridge and Chukar partridge metaphases. The aim of this fluorescence *In Situ* Hybridization (FISH) is to confirm rearrangement events and individually identify each pair of microchromosomes (work in progress). This study shows that, despite the importance of molecular investigation, banding cytogenetics is still an important step that provides basic knowledge on evolution of avian karyotypes.

## Funding

The present work has received financial support from the Ministère de l’Aménagement du Territoire et de l’Environnement MATE (project 223), Ministère de l’Enseignement Supérieur et de la Recherche Scientifique MESRS (project 209), Ministère de l’Intérieur, in the framework of Post Graduation Specialized: Empreintes génétiques en pratique judiciaire.
